# Utility and advantage of the unroofing technique for gastrointestinal subepithelial tumors: A multicenter retrospective cohort study

**DOI:** 10.1002/deo2.332

**Published:** 2024-01-19

**Authors:** Masashi Yamamoto, Tsutomu Nishida, Ryotaro Uema, Takashi Kanesaka, Hiroyuki Ogawa, Shinji Kitamura, Hideki Iijima, Kengo Nagai, Shusaku Tsutsui, Masato Komori, Katsumi Yamamoto, Yoshiki Tsujii, Yoshito Hayashi, Tetsuo Takehara

**Affiliations:** ^1^ Department of Gastroenterology Toyonaka Municipal Hospital Osaka Japan; ^2^ Department of Gastroenterology and Hepatology Osaka University Graduate School of Medicine Osaka Japan; ^3^ Department of Gastrointestinal Oncology Osaka International Cancer Institute Osaka Japan; ^4^ Department of Gastroenterology Nishinomiya Municipal Central Hospital Hyogo Japan; ^5^ Department of Gastroenterology Sakai City Medical Center Osaka Japan; ^6^ Department of Gastroenterology Osaka Police Hospital Osaka Japan; ^7^ Department of Gastroenterology Suita Municipal Hospital Osaka Japan; ^8^ Departments of Gastroenterology and Hepatology Itami City Hospital Hyogo Japan; ^9^ Department of Gastroenterology Hyogo Prefectural Nishinomiya Hospital Hyogo Japan; ^10^ Department of Gastroenterology Japan Community Healthcare Organization Osaka Hospital Osaka Japan

**Keywords:** gastrointestinal stromal tumor, mucosal incision‐assisted biopsy, subepithelial tumor, submucosal tumor, unroofing technique

## Abstract

**Background and aim:**

Various techniques for direct biopsy from gastrointestinal subepithelial tumors (SETs) have been reported, although no standard method has been established. A common feature of these techniques is the removal of overlaying mucosa to enable direct biopsies from the SETs. These methods have been synthesized under the collective term “unroofing technique”. We conducted a multicenter retrospective study to assess its efficacy and identify potential complications.

**Methods:**

This study was conducted in 10 hospitals and involved all eligible patients who underwent unroofing techniques to obtain biopsies for gastrointestinal SETs between April 2015 and March 2021. The primary endpoint was the diagnostic accuracy of the unroofing technique, and the secondary endpoints were the incidence of adverse events and the factors contributing to the accurate diagnosis.

**Results:**

The study included 61 patients with 61 gastrointestinal SETs. The median tumor size was 20 mm, and the median procedure time was 38 min, with 82% successful tumor exposure. The rate of pathological diagnosis was 72.1%. In 44 patients with a pathological diagnosis, two showed discrepancies with the postresection pathological diagnosis. No factors, including facility experience, organ, tumor size, or tumor exposure, significantly affected the diagnostic accuracy. There was one case of delayed bleeding and two cases of perforation.

**Conclusion:**

The diagnostic yield of the unroofing technique was acceptable. The unroofing technique was beneficial regardless of institutional experience, organ, tumor size, or actual tumor exposure.

## INTRODUCTION

Subepithelial tumors (SETs) are frequently encountered during routine endoscopies, with a detection rate of 0.36% to 0.76%.^1^, ^2^ SETs include various diseases ranging from leiomyomas, schwannomas, and ectopic pancreas to malignant tumors, such as gastrointestinal stromal tumors (GISTs) and neuroendocrine tumors (NETs). However, gastrointestinal SETs are difficult to diagnose pathologically because they are covered by normal mucosa. The European Society of Gastrointestinal Endoscopy (ESGE) guidelines suggest that endoscopic resection is an option for gastric SETs smaller than 20 mm to avoid unnecessary follow‐up in cases of diagnosis failure.^3^

Regarding the diagnostic method, endoscopic ultrasonography (EUS) is a valuable tool for SETs, enabling visualization of internal echogenicity, echotexture, and a layer of the tumor origin. However, the diagnostic yield of EUS is low,^4^, ^5^, ^6^ and interobserver agreement of EUS findings is often unfavorable.^7^ Recently, artificial intelligence has demonstrated the potential to increase the diagnostic accuracy of EUS by 20%–30%.^8^ However, it remains very important to obtain a pathological diagnosis to develop adequate strategies.

EUS‐guided fine‐needle aspiration cytology (EUS‐FNA) or EUS‐guided fine‐needle aspiration biopsy (EUS‐FNB) is a common method for obtaining tissue samples,^9^, ^10^, ^11^ but it requires convex EUS, which is not available in all institutions. Additionally, convex EUS has difficulty reaching the jejunum, ileum, and colon. It also has the limitation of low diagnostic yield in small SETs. The diagnosis rate of EUS‐FNA is reported to be as high as 88%–97% for tumors with a diameter of 20 mm or larger but as low as 53.9%–71.4% for tumors less than 20 mm in diameter due to technical difficulty.^12^, ^13^, ^14^, ^15^

The diagnosis rate of endoscopic boring biopsy is reportedly 25%–65% for SETs, as it often lacks direct visualization of the tumor itself.^16^, ^17^ Thus, various methods for tissue acquisition after removing overlying mucosa have been developed, including mucosal incision‐assisted biopsy (MIAB),^14^, ^15^ bloc biopsy with a mucosal flap,^18^ single‐incision needle‐knife biopsy (SINK),^19^ and others.^20^, ^21^, ^22^, ^23^ The American College of Gastroenterology guidelines endorse the unroofing technique when EUS‐FNA or FNB yields nondiagnostic results, paralleling ESGE guideline recommendations that regard MIAB and EUS‐FNB equally as equally viable options.^3^, ^24^ However, previous studies were typically single‐center or involved a limited number of institutions, leaving the method unstandardized. The unifying principle among these techniques is the excision of overlying mucosa to enable biopsies from the SETs. Therefore, it is feasible to consolidate these techniques under the encompassing term “unroofing technique”.

The diagnostic rate of the unroofing technique has been reported to be 77%–100%. Most of these reports were on gastric SETs. The analysis of the relationship between tumor size and diagnostic yield showed no difference in diagnostic efficacy, whereas organ‐specific and interinstitutional studies are still lacking. In addition, tumor exposure was reported to contribute to the success of tissue harvesting during the unroofing technique.^25^ However, the importance of tumor exposure in achieving a pathological diagnosis has yet to be definitively established because of the single‐center survey, which was limited to gastric lesions. To address these points, we conducted an exploratory utility study of the unroofing technique in a multicenter retrospective study of SETs in the entire gastrointestinal tract.

## METHODS

### Study design

This multicenter retrospective study was conducted at 10 hospitals in Japan, including one university hospital, one cancer center, two general hospitals, and six municipal hospitals. The study included all eligible patients who underwent the unroofing technique for gastrointestinal SETs between April 2015 and March 2021.

Patient data were collected from electronic medical records, and representative endoscopic static images were selected for central review of tumor exposure. We collected data including patient background information (sex, age, hemoglobin level, platelet count, prothrombin time, activated partial thromboplastin time, and antithrombotic medication details), endoscopic and EUS findings (tumor location, size, growth pattern, surface pattern, EUS layer, echo texture, and internal echogenicity), details of the unroofing technique procedure (device used, technique used, procedure time, number of biopsy specimens, adverse events, and histologic results) and institutional data (number of unroofing techniques during the enrollment period). Additional information on surgery and follow‐up after the unroofing technique procedure was collected (date of resection, final pathologic diagnosis, follow‐up duration, date of last endoscopy, tumor diameter change, date of last visit, and survival/death).

**FIGURE 1:**
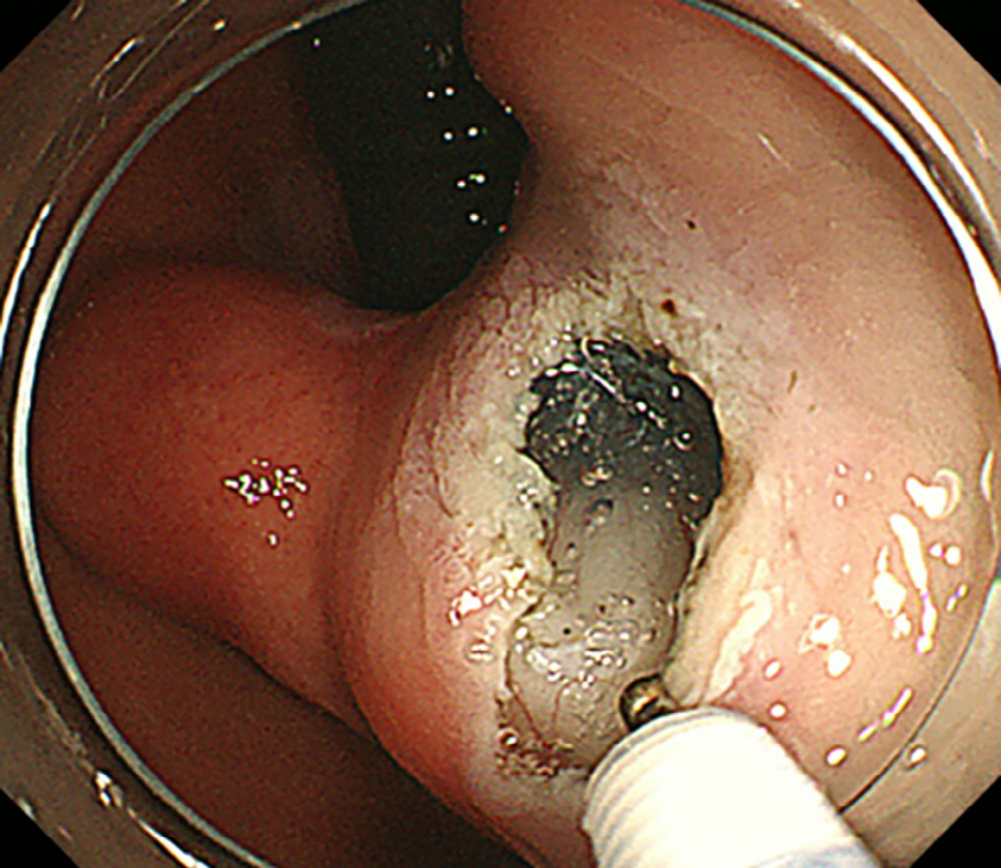
The overlying mucosa was removed to expose the surface of the subepithelial tumor. Cutting the mucosal and submucosal layers enables the subepithelial tumor to be visible.

The unroofing technique was defined as an endoscopic forceps biopsy after detaching the overlying mucosal and submucosal layers from the tumor to expose the tumor. Tumor exposure was defined as the endoscopic visibility of the tumor below the mucosal and submucosal layers and was determined with static figures by the consensus judgment of two expert endoscopists (Masashi Yamamoto and Ryotaro Uema). Figure 1 shows representative pictures of tumor exposure. The devices used and the procedure used were not limited. To preclude boring biopsy, we excluded cases in the outpatient setting and registered only hospitalized cases.

An accurate diagnosis was defined as a case in which the unroofing technique achieved a histopathological diagnosis of the tumor without discrepancy from the postresection diagnosis or a case in which the unroofing technique showed a result of no tumor in agreement with the postresection diagnosis. Diagnostic accuracy was defined as the rate of accurate diagnosis.

### Outcomes

The primary endpoint was the diagnostic accuracy of the unroofing technique, and the secondary endpoints were the incidence of adverse events and the factors contributing to the accurate diagnosis of the unroofing technique.

### Statement of ethics

The study was approved by the institutional review board of Osaka University and each participating facility and was performed in accordance with the Declaration of Helsinki. Patient consent was obtained using the opt‐out method on a designated website.

### Statistical analysis

Continuous variables are presented as the median and interquartile range. Categorical variables are presented as frequencies (%). Statistical significance was determined using the *χ*2 Fisher's exact test, with a *p*‐value less than 0.05 considered statistically significant. All statistical analyses were performed using JMP statistical software (ver. 16.20; SAS Institute Inc.).

## RESULTS

The study included 61 patients with 61 lesions who underwent the unroofing technique between April 2015 and March 2021. The median age was 59 years, and 41% of patients were male. Baseline laboratory data are shown in Table 1. Five patients were taking antithrombotic medications, including three on aspirin monotherapy and two on cilostazol monotherapy. Unroofing techniques for gastrointestinal SETs were performed in all portions of the gastrointestinal tract except the jejunum and ileum. The majority of gastrointestinal lesions were in the stomach (65.6%). The median tumor diameter was 20 mm, with four patients showing extramural growth. Before the biopsy, 57 patients underwent EUS, of which 68.4% were in the fourth layer and 29.8% were in the second to third layers. A total of 86.2% of cases were hypoechoic on EUS echotexture (Table 1).

**TABLE 1:** Patient characteristics of the unroofing technique.

Characteristics	*n* = 61
Age, median (IQR)	59 (46.5, 72)
Sex, men, *n* (%)	25 (41.0)
Baseline Laboratory data	
Hemoglobin, median (IQR) (g/dl)	13.9 (12.9, 14.6)
Platelet count, median (IQR) (10^10^/L)	23.7 (18.9, 30.1)
PT‐INR, median (IQR)	1.01 (0.95, 1.05)
APTT, median (IQR) (s)	30 (28, 32.8)
Antithrombotic agent use, *n* (%)	5 (8.3)
Anticoagulant, *n* (%)	0 (0)
Antiplatelet agent, *n* (%)	5 (8.3)
Aspirin	3 (5.0)
Cilostazol	2 (3.3)
Tumor location	
Esophagus, *n* (%)	7 (11.4)
Stomach, *n* (%)	40 (65.6)
Duodenum, n (%)	6 (9.8)
Colon, *n* (%)	4 (6.6)
Rectum, *n* (%)	4 (6.6)
Tumor size, median (IQR) (mm)	20 (15, 29.5)
Tumor growth, extramural, *n* (%)	4 (6.6)
Tumor surface, regular, *n* (%)	53 (91.4)
EUS layer of tumor	
2nd–3rd layer, *n* (%)	17 (29.8)
4th layer, *n* (%)	39 (68.4)
Others	1 (1.8)
EUS echotexture, hypoechoic, *n* (%)	50 (87.7)
EUS internal echogenicity, regular, *n* (%)	42 (73.7)

Abbreviations: APTT, activated partial thromboplastin time; EUS, endoscopic ultrasonography; IQR, Interquartile range; PT‐INR, prothrombin time‐international normalized ratio; SET, subepithelial tumor.

Electrosurgical knives (needle knives or ESD knives) were used in 57 cases (93.4%) for the unroofing technique, with snares and hot biopsy forceps used in six cases. Fifty cases (82.0%) were judged to achieve successful tumor exposure by central judgment. The median number of biopsies performed was six, and the median duration of the procedure, from the initiation of mucosal incision or submucosal injection to completion of tissue collection or clip closure, was 38 min. Tissue acquisition was successful in 60 of 61 cases (98.4%), except for one patient who withdrew due to intraoperative perforation. Of the 44 cases (72.1%) that led to a pathologic diagnosis, 19 (31.1%) patients underwent surgery, resulting in two cases with discrepancies between the unroofing technique and pathological surgical results. (Table 2). The first case involved a 16 mm gastric SET originating from the fourth layer. Successful exposure through the unroofing technique led to an initial diagnosis of leiomyoma. Subsequent surgical resection, however, yielded a final pathological diagnosis of fibroma. In the second case, a 20 mm duodenal SET originating from the third layer was successfully exposed, revealing small round cells with immunohistochemical CD56 positivity, suggestive of a duodenal NET. Surgical resection later clarified the diagnosis as pancreatic heterotopia, Heimlich type I, characterized by a pronounced presence of islets of Langerhans.

**TABLE 2:** Results of the unroofing technique.

Devices used for exposing tumor	
Electrosurgical knife, *n* (%)	57 (93.4)
Hot biopsy forceps, *n* (%)	6 (9.8)
Snare, *n* (%)	6 (9.8)
Conventional biopsy forceps, *n* (%)	0 (0)
Procedure time, median (IQR) (min)	38 (21.5, 68)
Success of tumor exposure, *n* (%)	50 (82.0)
Success of tissue acquisition, *n* (%)	60 (98.4)
Number of tissue samples, median (IQR) (min)	6 (4, 9)
Adverse events	
Intraoperative massive bleeding, *n* (%)	0 (0)
Delayed bleeding, *n* (%)	1 (1.6)
Perforation, *n* (%)	2 (3.3)
Others, *n* (%)	0 (0)
Obtainment of pathological diagnosis for SET, *n* (%)	44 (72.1)
Gastrointestinal stromal tumor	19
Leiomyoma	11
Ectopic pancreas	4
Schwannoma	3
Neuroendocrine tumor	2
Others	5
Discrepancy with postresection pathological diagnosis, *n* (%)	2

Abbreviations: IQR, Interquartile range; SET, subepithelial tumor.

A pathological diagnosis was not obtained in 17 of 61 (27.9%) unroofing technique cases. The tumor exposure rate in these undiagnosed cases was 88.2% (15 cases). Nine of the 17 patients underwent resection for suspected malignancy, and the other eight patients were followed for a median of 1539 days. Of the nine resected cases, three resulted in GISTs, one resulted in gastric adenocarcinoma with lymphoid stroma, three resulted in benign tumors, and one resulted in no tumor. Two of three cases with GIST were successful in tumor exposure. In the eight unresected cases, no cases grew or changed the shape of SETs; three cases (37.5%) had a reduction in tumor size (Table 3). Of the three shrunken cases, the first was a gastric subepithelial tumor 14 mm in diameter. The unroofing technique collected fibrotic tissue, and the tumor shrank after 540 days of follow‐up. The second case was a colorectal submucosal tumor 10 mm in diameter. The unroofing technique samples showed submucosal tissue with lymphocyte infiltration. Follow‐up endoscopy revealed tumor shrinkage during the observation period of 1539 days. The third case was an esophageal subepithelial tumor 25 mm in diameter. The tissue obtained by the unroofing technique was submucosal tissue and smooth muscle. The tumor endoscopically disappeared after 1247 days.

**TABLE 3:** Clinical course of cases with failed pathological diagnosis.

Cases with failed diagnosis	*n* = 17
Resection, *n* (%)	9 (52.9)
Tumor size, median (IQR) mm	22 (20, 33.5)
Final diagnosis after resection	
Gastrointestinal stromal tumor	3
Leiomyoma	1
Mesenchymal tumor	1
Implantation cyst	1
Adenocarcinoma with lymphoid stroma	1
No tumor	1
Unknown (resected at another facility)	1
Follow up, *n* (%)	8 (50.0)
Tumor size, median (IQR) median	21 (15.3, 29.8)
Endoscopic follow‐up period, median (IQR) days	764 (196, 1416.5)
Clinical follow‐up period, median (IQR) days	1373 (930.8, 1707.5)
Change in tumor size	
Enlargement	0
No change	5
Shrinkage	3

Abbreviations: GIST, gastrointestinal stromal tumor; IQR, Interquartile range.

This study evaluated factors contributing to accurate diagnosis with the unroofing technique, including facility experience with the unroofing technique, tumor location, tumor size, EUS layer, EUS echotexture, procedure time, number of tissue samples, and tumor exposure. Our results showed that none of these factors affected the success or failure of the accurate diagnosis (Table 4). The diagnostic accuracies for tumors <20 mm and ≥20 mm were 76.9% and 65.7%, respectively (Table 5). The diagnostic accuracy at institutions with less than 10 experiences was 70.4%, and at institutions with more than 10 experiences, the rate was 70.6% (Table 6).

**TABLE 4:** Univariate analysis of factors contributing to the accurate diagnosis of the unroofing technique.

	Univariate logistic analysis		
Variables	Odds ratio	95% CI	*p*‐value
Facility experience, ≥10 cases, yes	1.01	0.33–3.06	0.9852
Age, ≥60 years, yes	0.70	0.23–2.10	0.5191
Tumor location, esophagus, yes	2.83	0.32–25.4	0.3046
Tumor location, stomach, yes	0.9	0.28–2.89	0.8591
Tumor location, duodenum, yes	2.24	0.24–20.6	0.4454
Tumor location, colon/rectum, yes	0.36	0.08–1.63	0.1897
Tumor size, ≥20 mm, yes	0.58	0.18–1.81	0.3387
Tumor growth, extramural, yes	0.39	0.33–19.8	0.3733
EUS layer, the 4th layer, yes	0.98	0.28–3.40	0.9734
EUS echotexture, hypoechoic, yes	2.85	0.62–13.1	0.1830
Procedure time, ≥38 min, yes	0.76	0.43–3.96	0.6319
Number of tissue samples, ≥6, yes	0.40	0.75–8.18	0.1234
Tumor exposure, yes	0.47	0.09–2.44	0.3449

Abbreviations: CI, confidence interval; EUS, endoscopic ultrasonography.

**TABLE 5:** Diagnostic rate of the unroofing technique by tumor diameter.

	20 mm>	20 mm≤	
Tumor diameter	*n* = 26	*n* = 35	*p*‐value
Pathological diagnosis, *n* (%),	21 (80.8)	23 (65.7)	0.1887
Accurate diagnosis, *n* (%)	20 (76.9)	23 (65.7)	0.3387

**TABLE 6:** Diagnostic rate of the unroofing technique by facility experience.

	10 cases>	10 cases≤	
Facility experience	*n* = 27	*n* = 34	*p*‐value
Pathological diagnosis, *n* (%),	20 (74.1)	24 (70.6)	0.7626
Accurate diagnosis, *n* (%)	19 (70.4)	24 (70.6)	0.9852

Among the 35 cases diagnosed under tumor exposure, the majority were GIST, accounting for 15 cases, followed by leiomyoma, schwannoma, and ectopic pancreas. Among nine cases diagnosed without tumor exposure, GIST was the most common for four cases, while leiomyoma, schwannoma, and ectopic pancreas were found in the remaining cases (Table 7).

**TABLE 7:** Pathological diagnosis of unroofing technique with or without tumor exposure.

Pathological diagnosis with tumor exposure, *n* (%)	35 (70.0)
GIST	15
Leiomyoma	10
Ectopic pancreas	3
Schwannoma	2
Neuroendocrine tumor	1
Others	3
Pathological diagnosis without tumor exposure, *n* (%)	9 (81.8)
GIST	4
Leiomyoma	1
Ectopic pancreas	1
Schwannoma	1
Neuroendocrine tumor	1
Others	1

Abbreviation: GIST, gastrointestinal stromal tumor.

Perforations were observed in two out of the 61 cases using the unroofing technique (3.3%). Both cases involved SET originating from the fourth layer of the gastric wall, measuring 22 and 29 mm, respectively. In the two cases, an ESD scalpel was used to make an incision at the lateral side rather than the top of the SETs, resulting in perforations due to damage to the peritumoral muscle layer after a wider incision. Both patients underwent clip sutures and improved conservatively without surgical resection and were discharged on the fifth and sixth days after the procedure. In contrast, no perforations occurred in all cases in which the unroofing techniques were performed near the tumor's apex.

## DISCUSSION

Our study enrolled 61 cases of the unroofing technique in the whole gastrointestinal tract. The diagnostic accuracy of the unroofing technique was 72.1%. Similar to previous reports,^12^, ^13^, ^14^, ^15^ tumor size did not affect the diagnostic accuracy, and no significant relationship existed between the target organs and the results. EUS‐FNA/FNB is supposed to be difficult in some cases, such as small SETs and colonic SETs. Our results indicated that the unroofing technique is a feasible alternative to EUS‐FNA/FNB in the entire gastrointestinal tract.

We collected endoscopic pictures during the unroofing technique and judged success or failure in tumor exposure under the central view of two experts. Unlike the previously reported results, tumor exposure did not significantly improve the diagnostic accuracy, even when limited to tumors originating from the fourth layer and by organ (data not shown). The diagnostic utility of the unroofing technique seemed independent of tumor exposure. Notably, two gastric GIST cases did not achieve a diagnosis through biopsy, despite successful exposure. This is likely due to the firmness of the tumor and inadequate specimen collection for standard biopsy forceps. This underscores the potential need for specialized biopsy forceps.

Our study noted two cases of pathological discrepancies between the unroofing technique and surgical resection, despite successful tumor exposure. First, smooth muscle and fibrotic tissue led to a preliminary leiomyoma diagnosis, in which surgical resection was corrected to fibroma. This implies that the harvested smooth muscle was from the overlying muscularis mucosa. The second case involved CD56‐positive cell identification from the mass, initially indicating NETs, but resection confirmed ectopic pancreas, Heimlich type I. This highlights the difficulty of differentiation between NETs and pancreatic heterotopia. These results emphasize the necessity of a comprehensive diagnosis, not just a biopsy. Two perforations (3.3%) occurred when incisions were made tangentially to tumors larger than 20 mm in diameter, despite submucosal injections aimed at preventing perforation. Bleeding and edema obscured the tumor margins, leading to inadvertent damage to the peritumoral muscle layer. In the unroofing technique, the approach from the tumor apex is the safest, with the least risk of injury to the muscle layer. In our study, there were no cases of perforation when the unroofing technique took place close to the tumor apex. Based on the above points, even when a tangential approach is unavoidable, the risk of perforation can be avoided by keeping the approach as close to the apex as possible. When unroofing is needed near the muscle layer, employing the submucosal tunnel method ensures clear identification of the muscle layer, or the SINK method may be advantageous because of making incisions before bleeding or edema. Clear delineation of the tumor‐muscle interface is essential; therefore, flat SETs, with poorly defined borders relative to the surrounding muscle, are contraindicated for unroofing. If the safety of this procedure can be assured, the unroofing technique can become an outpatient procedure. Therefore, the analysis of adverse events is informative and should be verified prospectively in the future.

We enrolled 10 participating hospitals, including a university hospital, a cancer center, general hospitals, and municipal hospitals, to analyze the relationship between proficiency in the unroofing technique and the success rate of obtaining an accurate diagnosis. Our analysis revealed that the number of experiences at each facility did not significantly affect the diagnostic accuracy of the unroofing technique. Many hospitals preferred electrosurgical knives, reflecting endoscopists’ comfort and experiences with these tools from endoscopic submucosal dissection. The key is using devices with which clinicians are most familiar to ensure adequate unroofing. Consequently, the unroofing technique is relatively easy to introduce to a hospital that provides regular endoscopic procedures.

This study has several limitations. First, the small number of patients enrolled in the study necessitates an increase in patient enrollment for multivariate analysis. Second, since this is a retrospective study, enrollment was restricted to inpatients only to avoid contamination of boring biopsy cases. However, the unroofing technique using biopsy forceps has been performed in outpatient settings. A prospective study is needed to verify our results.

In conclusion, the unroofing technique is acceptable for diagnosing subepithelial tumors if the approach is kept close to the apex to avoid perforation. It is relatively easy to introduce and obtain tissue samples in the entire gastrointestinal tract. Prospective studies with larger sample sizes are needed to validate the safety and efficacy of this technique.

## CONFLICT OF INTEREST STATEMENT

None.
